# The gut microbiota-metabolite-target axis in insomnia: network pharmacology and gut microbiota profiling insights into pathogenesis and intervention with Cinnamomi Cortex-Gardeniae Fructus

**DOI:** 10.3389/fmicb.2026.1830736

**Published:** 2026-06-29

**Authors:** Jiaquan Yuan, Dongyang Wu, Haoyang Xu, Junyao Jiao, Chen Yang, Xiucai Wu, Weifeng Liu, Erwei Hao

**Affiliations:** 1 Gradute School of Guangxi University of Chinese Medicine, Nanning, China; 2 Guangxi Key Laboratory of Efficacy Study on Chinese Materia Medica, Guangxi University of Chinese Medicine, Nanning, China; 3 Guangxi International Zhuang Medicine Hospital, Nanning, China

**Keywords:** Cinnamomi Cortex, Gardeniae Fructus, gut microbiota, gut-brain axis, insomnia, metabolites, network pharmacology

## Abstract

**Objective:**

To investigate the mechanism underlying the association between gut microbiota metabolites and insomnia, focusing on the “gut microbiota-metabolite-target” axis. The Cinnamomi Cortex-Gardeniae Fructus (CC-GF) combination was used as an intervention.

**Methods:**

Network pharmacology was applied to predict insomnia-related gut microbiota metabolites and their targets. A PCPA-induced rat insomnia model was established, and gut microbiota dysbiosis was validated using 16S rRNA sequencing. Core gut microbiota were identified, and core targets were screened via protein–protein interaction (PPI) network analysis and multi-algorithm ranking. A “core microbiota-metabolite-core target” network was constructed, and binding affinities were verified using molecular docking and molecular dynamics simulations.

**Results:**

Twenty-eight targets were predicted, and CASP3, NFE2L2, HDAC1, and CCND1 were identified as core targets related to oxidative stress, apoptosis, epigenetic regulation, and circadian disturbance. In the insomnia model, the beneficial genera *Bifidobacterium* and *Bacteroides* were decreased, whereas the harmful genera *Ruminococcus* and *Blautia* were increased. Nine key metabolites were identified; folic acid exhibited the strongest binding affinity to the core targets (average −8.83 kcal/mol) and was defined as the core metabolite. CC-GF reversed these microbiota alterations, confirming the regulatory role of this axis.

**Conclusion:**

This study elucidates the “gut microbiota-metabolite-target” axis in insomnia and highlights the “*Bifidobacterium*-folic acid-target” pathway as a core mechanism. CC-GF intervention provides experimental evidence supporting this axis as a target for insomnia treatment.

## 1 Introduction

Insomnia is a common sleep disorder characterized by difficulty falling asleep, maintaining sleep, or early awakening, significantly affecting daily life, physical and mental health, and work efficiency. Epidemiological studies show that approximately 10% of the adults suffers from chronic insomnia disorder, while about 20% of adults experience occasional or transient insomnia symptoms, with higher prevalence in women and the elderly (Morin and Jarrin, 2022). The etiology of insomnia is complex and involves various biological and psychological factors. Studies have shown that chronic insomnia may be closely related to an overactive state of the central nervous system, particularly the role of neurotransmitter systems in regulating sleep-wake cycles (Riemann et al., 2010). At the same time, the persistence of insomnia is closely associated with cognitive biases, such as excessive worry about sleep and negative expectations about insomnia. This indicates a close link between psychological factors (Harvey, 2002). Currently, pharmacological treatment is one of the most commonly used methods for managing insomnia. Commonly used drugs include benzodiazepines (e.g., diazepam, lorazepam) and non-benzodiazepine drugs (e.g., zolpidem, eszopiclone). These medications exert their sedative and anxiolytic effects by enhancing the action of γ-aminobutyric acid (GABA-A) receptors in the brain, effectively reducing sleep onset time and improving sleep quality (Sateia et al., 2017; Morin and Benca, 2012). However,these drugs are often associated with the risk of adverse effects, including cognitive impairment, tolerance, and dependence (Brandt and Leong, 2017).

The gut microbiota is a new frontier in health and disease (Dogra et al., 2020). The gut-brain axis plays an important role in regulating sleep. The gut communicates bidirectionally with the brain through the vagus nerve, endocrine system, and metabolites produced by the gut microbiota. These signals have a potential impact on sleep patterns and quality (Strandwitz, 2018). The balance of the gut microbiota has a significant impact on sleep regulation. Dysbiosis of the gut microbiota can disrupt sleep patterns and quality by altering the levels of metabolites and neurotransmitters (such as serotonin), thereby interfering with the signaling in the central nervous system. This mechanism provides a new perspective for understanding the pathogenesis of insomnia (Lin et al., 2024). However, the specific regulatory role of the gut microbiota-metabolite-target axis in insomnia remains poorly understood.

Traditional Chinese Medicine (TCM) has certain advantages in the treatment of insomnia through the synergistic effects of multiple components and targets (Zhou et al., 2017). Cinnamomi Cortex (CC) is commonly used in traditional Chinese medicine and also as a traditional spice, belonging to the category of food-medicine homologous species. Modern pharmacological studies have confirmed its neuroprotective effects. Its metabolites have been shown to regulate the PKA-CREB signaling pathway, increasing the levels of brain-derived neurotrophic factor (BDNF) and neurotrophin-3 in the brain, potentially influencing neural function. This provides support for its potential role in sleep-related regulation (Liu et al., 2021; Zhang et al., 2019). Gardeniae Fructus (GF) is a commonly used herb in traditional Chinese medicine. It also belongs to the category of food-medicine homologous species (Tian et al., 2022). Geniposide, the main active component of Gardeniae Fructus, exhibits pharmacological effects such as neuroprotection, anti-inflammatory, and antioxidant properties in various biological processes. These effects provide a potential pharmacological basis for its role in regulating central nervous system function (Liu et al., 2022).

This study aims to systematically elucidate the association between gut microbiota and their metabolites with insomnia, as well as the underlying molecular mechanisms. Using network pharmacology, molecular docking, animal experiments, and gut microbiota sequencing, the food-medicine homologous herbal combination CC-GF was employed as a research model to explore whether it ameliorates insomnia by correcting specific gut microbiota dysbiosis and subsequently regulating related metabolites. This research approach provides a feasible therapeutic strategy for intervening in insomnia from the perspective of gut microbiota metabolites.

## 2 Materials and methods

### 2.1 Animal experimental materials and reagents

Gardeniae Fructus combined granules (Batch No. 17364214; equivalent original drug: 10 g/bag) and Cinnamomi Cortex combined granules (Batch No. 17364240; equivalent original drug: 4 g/bag) were provided by Jiangsu Tianjiang Pharmaceutical Co., Ltd. (Wuxi, China). 4-Chloro-DL-phenylalanine (PCPA; Batch No. 47LCC-LI; 25 g) was purchased from TCI Chemicals (Shanghai) Co., Ltd. (Shanghai, China). Sodium carboxymethyl cellulose (CMC-Na; Batch No. B2215321; 500 g) was obtained from Shanghai Aladdin Biochemical Technology Co., Ltd. (Shanghai, China). Sodium chloride injection (0.9%; Batch No. 2110190724; 500 ml/bag) was provided by Qilu Pharmaceutical Co., Ltd. (Jining, China). A 4% paraformaldehyde (PFA) solution (Batch No. 22360481; 500 ml) was purchased from SaiGuo Biotechnology Co., Ltd. (Guangzhou, China). A 10% sodium pentobarbital solution (Batch No. AA3010) was obtained from Beijing Solabio Technology Co., Ltd. (Beijing, China). Ultra-pure water was prepared using the Milli-Q Reference water purification system (Merck Millipore, USA).

### 2.2 Drug preparation

#### 2.2.1 Preparation of Cinnamomi Cortex and Gardeniae Fructus combined solution

The Cinnamomi Cortex and Gardeniae Fructus combined granules were mixed in a 6:1 ratio of original drug mass, and dissolved in ultra-pure water to prepare the Gardenia-Cinnamon combined solution (with a concentration of 0.735 g/mL of original drug). The solution was stored at −20 °C for future use.

#### 2.2.2 Preparation of PCPA suspension (40 mg/mL)

Twenty grams of sodium carboxymethyl cellulose (CMC-Na) were dissolved in 400 mL of physiological saline (0.9% NaCl) and continuously heated with stirring to obtain a 0.5% (w/v) CMC-Na solution, which was stored at room temperature for future use. To prepare the PCPA suspension, 30 mL of the 0.5% CMC-Na solution was gradually added to 1.2 g of PCPA powder in a 50 mL centrifuge tube. The mixture was vortexed thoroughly to form a uniform particle suspension, resulting in a 40 mg/mL PCPA suspension. The final suspension was stored at 4 °C until use.

### 2.3 Experimental animals and PCPA-induced insomnia model

#### 2.3.1 Ethical approval and animal handling

This study was approved by the Animal Ethics Committee of Guangxi University of Chinese Medicine (Approval No. 2021DW172). Thirty-two male Sprague-Dawley (SD; SPF grade; initial weight 180 ± 20 g) were provided by Hunan SJA Laboratory Animal Co., Ltd. [Certificate No. SCXK(Xiang)2019-0004]. The animals were housed at the Experimental Animal Center of Guangxi University of Chinese Medicine (Nanning, China) under controlled environmental conditions: temperature of 25 °C ± 2 °C, relative humidity of 50% ± 10%, and a 12-h light/dark cycle. During the 7-day acclimation period, the animals had free access to food and water, and all experimental procedures complied with national animal welfare regulations.

#### 2.3.2 PCPA-induced insomnia model

Based on the literature and previous studies by the research team, a PCPA-induced insomnia rat model was established. PCPA (400 mg/kg) was administered via intraperitoneal injection for 3 consecutive days. The control group received an equal volume of physiological saline for 4 consecutive days.

### 2.4 Experimental groups, intervention, and sample collection

Eight healthy rats were used as the control group, while the PCPA-induced rats were randomly divided into three treatment groups (*n* = 8 per group): the model control group, the DZP positive control group (2 mg/kg), and the CC-GF decoction group (2.5 g/kg original drug equivalent, representing 10 times the clinical human dose). All groups were orally gavaged once daily at a fixed morning time for 7 consecutive days (20 mL/kg dosing volume). During the treatment period, the blank control group and the model control group received an equal volume of physiological saline. Prior to the final administration, all rats underwent 12 h of fasting, with unlimited access to water. One hour after administration, the animals were anesthetized by intraperitoneal injection of sodium pentobarbital (45 mg/kg). After confirming successful anesthesia by the loss of the righting reflex and absence of pain response when the rats were placed in a supine position, dissection was initiated. During dissection, intestinal contents were collected under sterile conditions and stored at −80 °C for gut microbiota analysis. Following specimen collection, the animals were euthanized while under anesthesia through exsanguination from the abdominal aorta. This procedure, along with all animal handling in this study, was performed in strict accordance with the ARRIVE guidelines and national ethical standards to minimize suffering.

### 2.5 Behavioral tests

#### 2.5.1 Open field test

One day prior to testing, rats were acclimatized to the experimental environment for 60 min. On the test day, each rat was sequentially placed at the same starting position within a square open-field arena (100 cm × 100 cm). Behavioral parameters including total distance traveled, average velocity, number of central zone entries, duration in central zone, and rearing frequency were recorded over a 5-min session. These metrics collectively evaluated Anmeidan's effects on spatial exploration ability and anxiety-/depression-like behaviors in insomnia model rats.

#### 2.5.2 Morris water maze procedure

Spatial cognitive function was assessed using the Morris water maze according to established methodologies. Twenty-four hours prior to testing, animals were transferred to the behavioral laboratory for environmental acclimatization. This was followed by three consecutive days of hidden platform training (one session daily), during which mice were required to locate a submerged platform within 60 s, with unsuccessful trials assigned the maximum latency of 60 s. On day 4, a probe trial was conducted: following platform removal, mice underwent a 120-s free swim while key spatial memory parameters were recorded, including latency to reach the target quadrant, number of platform location crossings, and percentage duration in the target quadrant.

### 2.6 Enzyme-linked immunosorbent assay (ELISA)

Serum levels of 5-HT and GABA were quantified using commercial ELISA kits (5-HT: Elabscience E-EL-0035; GABA: Jiancheng Bio H203). Thawed serum samples were centrifuged at 10,000 × g for 10 min at 4 °C, with supernatants diluted 1:5 in assay buffer. Following manufacturer protocols, 100 μL aliquots of standards and samples were loaded in duplicate onto pre-coated plates and incubated at 37 °C. After sequential additions of detection antibodies and enzyme conjugates with intermittent PBS-T washes, color development was initiated with TMB substrate and stopped with 2M H_2_SO_4_. Absorbance was measured at 450 nm (reference 570 nm), with concentrations calculated against standard curves and normalized to total protein content.

### 2.7 Microbial DNA extraction and 16S rRNA sequencing

After dissection, colon content samples were randomly collected from eight rats per group: blank control group, model group, and CC-GF intervention group. The total microbial DNA extraction was performed by Shenzhen BGI Genomics. Subsequently, the V3–V4 hypervariable region of the 16S rRNA gene was PCR amplified using fusion primers. PCR reactions were conducted with 30 ng of qualified genomic DNA as the template under optimized cycling conditions. The amplification products were purified using a magnetic bead purification system and re-suspended in elution buffer. The purified fragments were then barcoded and library construction was carried out. Quality control was performed using the Agilent 2100 Bioanalyzer to confirm that the fragment size distribution and library concentration met the required standards. Finally, the qualified libraries were sequenced based on the insert fragment size, and the resulting data were used for gut microbiota taxonomy annotation and abundance analysis.

### 2.8 Data sources and preprocessing

This study utilized several public databases to screen targets related to gut microbiota metabolites and insomnia. First, the target data for gut microbiota metabolites were obtained from the Similarity Ensemble Approach (SEA) platform (https://sea.bkslab.org/) and the Swiss Target Prediction (STP) platform (http://www.swisstargetprediction.ch/). Microbial metabolite-Host Gene data were sourced from: https://bio-computing.hrbmu.edu.cn/gutmgene/#/home. To further screen insomnia-related targets, the gene expression profile dataset GSE208668 (platform: GPL10904) was downloaded from the Gene Expression Omnibus (GEO) database. The dataset included a total of 42 peripheral blood mononuclear cell (PBMC) samples, comprising 17 patients with primary insomnia (7 males and 10 females) and 25 healthy controls (11 males and 14 females). Notably, the subjects in the GSE208668 dataset were predominantly elderly individuals. Given the lack of high-quality insomnia-related transcriptome data covering young and middle-aged populations in public databases, this dataset was still adopted for target screening in the present study, aiming to explore the common molecular mechanisms of insomnia.

By performing intersection analysis between the targets of gut microbiota metabolites and the Microbial metabolite-Host Gene data, we identified the main predicted targets through which gut microbiota metabolites regulate insomnia.

### 2.9 GO/KEGG enrichment analysis of predicted targets

To explore the biological functions of these key targets, we performed Gene Ontology (GO) enrichment analysis using Cluego in Cytoscape 3.10.2, which included Biological Process (BP) and Molecular Function (MF) categories. Additionally, Kyoto Encyclopedia of Genes and Genomes (KEGG) pathway enrichment was performed by integrating data from the DAVID database. Through KEGG enrichment analysis, important pathways associated with the predicted targets for the treatment of insomnia were identified and visualized.

### 2.10 PPI network analysis and core target screening

To identify key predicted targets in the treatment of insomnia, we first constructed a PPI network using the STRING database (https://string-db.org/), setting the species to “Homo sapiens” and a medium confidence threshold (>0.4). The PPI network was then imported into Cytoscape 3.10.2 for visualization and further network construction. To further screen core targets, we employed four algorithms (Degree, DMNC, MCC, and MNC) from the CytoHubba plugin for analysis, selecting the top 10 targets from each algorithm. Venn diagram analysis was used to identify the intersection, and the final core targets were determined. These core targets are considered to play a crucial role in the treatment of insomnia.

### 2.11 Core gene regulatory network construction and GO/KEGG enrichment analysis

To investigate the functional interactions of key genes, a gene-gene interaction (GGI) network was constructed using the GeneMANIA platform (https://genemania.org/). GO enrichment analysis, including Biological Process (BP) and Molecular Function (MF), was performed using Cluego in Cytoscape 3.10.2. Additionally, KEGG pathway enrichment was conducted by integrating data from the DAVID database to analyze the pathways associated with the targets.

### 2.12 “Gut microbiota-metabolites-substrate-core targets” network construction

To further explore the relationship between core targets and their corresponding gut microbiota metabolites, we used Cytoscape 3.10.2 to construct a direct network linking gut microbiota, metabolites, substrates, and core targets. The main metabolites and gut microbiota directly regulating the core targets were predicted. Subsequently, by comparing the key gut microbiota that were reversed by drug treatment in the insomnia mouse model, we identified the core gut microbiota.

### 2.13 Construction of the “core gut microbiota-metabolites-core targets” regulatory network and key metabolite screening

To further identify the metabolites that are directly regulated by the core gut microbiota and intervene with the core targets, we used Cytoscape 3.10.2 to construct a direct relationship map between the core gut microbiota, metabolites, and core targets. This rigorous analysis allowed us to accurately pinpoint the most critical metabolites within the network, thereby enhancing our understanding of their roles in biological systems.

### 2.14 Gene-miRNA, gene-TF network diagram

Transcription factors corresponding to the core genes were obtained using the JASPAR database (https://jaspar.elixir.no/). MicroRNA (miRNA) predictions were performed using the miRWALK (http://mirwalk.umm.uni-heidelberg.de) and miRDB (http://www.mirdb.org) databases. Common transcription factors and miRNAs targeting the core genes were identified, providing insights into potential regulatory interactions that may influence the pathogenesis of the disease.

### 2.15 Molecular docking

To analyze the binding modes and affinities between key compounds and core targets, molecular docking was performed in this study. First, the 3D structures of target compounds were obtained from the PubChem database (https://pubchem.ncbi.nlm.nih.gov/), and the corresponding crystal structures of core targets were downloaded from the Protein Data Bank (PDB; https://www.rcsb.org/). Preliminary docking analysis was conducted using the online tool CB-Dock 2 (https://cadd.labshare.cn/cb-dock2/php/index.php), followed by more precise molecular docking calculations using AutoDock Vina to assess the binding free energy of the complexes. Finally, the docking results were visualized in 3D using PyMOL software, providing a systematic interpretation of the interactions between the compounds and the targets.

### 2.16 Molecular dynamics simulation

In this study, molecular dynamics simulations were performed using GROMACS version 2025.1. The topology files for the ligands were generated using the CHARMM36 force field, including the TIP3P water model, and energy minimization was carried out using the Steepest Descent Method and the Conjugate Gradient Method. After energy minimization, a 100 ns simulation was conducted under NVT conditions, gradually heating the system to 300 K. Subsequently, the system was equilibrated under isothermal-isobaric (NPT) conditions. Finally, a 200 ns production run was performed for the molecular dynamics simulation. During the simulation, in addition to conventional analysis methods such as RMSD and SASA, we also performed Principal Component Analysis (PCA) to assess the system's stability and characterize its features under different states.

### 2.17 Statistical analysis

Statistical analysis was performed using SPSS 20.0 software (IBM, USA). All data were first tested for normality using the Shapiro–Wilk test (given the sample size of *n* = 8 per group, the Shapiro–Wilk test is more appropriate than the Kolmogorov–Smirnov test). Homogeneity of variances was assessed using Levene's test. For data that met both normality and homogeneity of variances, one-way ANOVA followed by Tukey's *post-hoc* test was used for multiple comparisons between groups. For data that did not meet normality or homogeneity of variances (even after transformation), the non-parametric Kruskal–Wallis test was applied, followed by Dunn's *post-hoc* test with Bonferroni correction. Student's *t*-test (or Welch's *t*-test when variances were unequal) was used for two-group comparisons where applicable. All statistical tests were two-tailed, and a *p*-value < 0.05 was considered statistically significant.

## 3 Results

### 3.1 . CC-GF ameliorates general conditions, behaviors, and neurotransmitter levels in PCPA-induced insomnia model rats

PCPA treatment significantly altered the states of rats. The Control group rats exhibited glossy and smooth fur, with no notable weight gain. Their feces were black and hard, and their cages remained dry. They maintained a normal mental state and displayed clear circadian rhythms. In contrast, the Model group showed matted and disheveled fur, weight loss, increased urine and fecal output, and grayish-white, soft stools. Their cages were moist, and they exhibited abnormal emotional behaviors, including social withdrawal, irritability, aggression, and heightened sensitivity to external stimuli, indicating successful modeling of insomnia in rats. Conversely, the DZP group and CC-GF group rats demonstrated gradual improvement in fur texture toward smoothness, along with steady weight gain. Their fecal color gradually turned black, and their mental state improved. Additionally, urine output decreased, showing no significant difference compared to the blank control group. These findings indicate that CC-GF can effectively improve the general condition of insomnia-induced rats.

**Figure 1:**
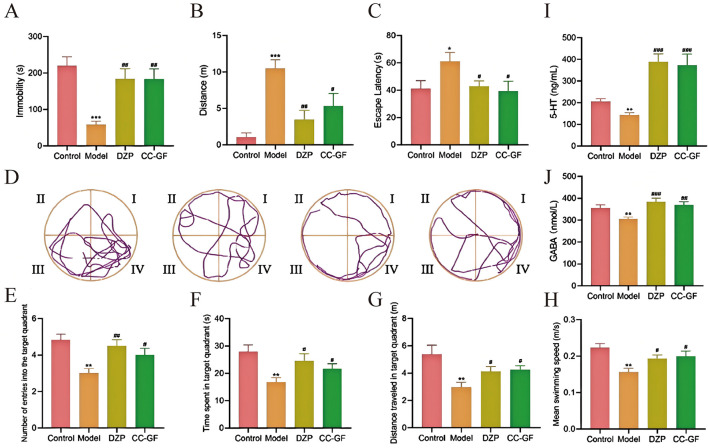
CC-GF ameliorates general conditions, behaviors, and neurotransmitter levels in PCPA-induced insomnia model rats. **(A)** Immobility of rats in each group in open field test during the open field test; **(B)** Total distance traveled of rats in each group during the open field test; **(C)** Escape latency of rats in each groups during the Morris water maze test; **(D)** Tracking via the water maze imaging system revealed distinct behavioral patterns; **(E)** Number of entries the target quadrant of rats in each group during the Morris water maze test; **(F)** Time spend in target quadrant of rats in each group during the Morris water maze test; **(G)** Distance traveled in target quadrant of rats in each group during the Morris water maze test; **(H)** Mean swimming speed in the target quadrant of rats in each group during the Morris water maze test; **(I)** Serum 5-HT levels of rats in each group; **(J)** Serum GABA levels of rats in each group. Data are presented as mean ± SEM, *indicates a significant difference between the Control group and the model group; ^#^indicates a significant difference between the model group and the DZP group, the CC-GF group. *p < 0.05, **p < 0.01, and ***p < 0.001 vs. the control group; ^#^*p* < 0.05, ^##^*p* < 0.01, and ^###^*p* < 0.001 vs. the model group.

Open Field Test (OFT): The OFT assessed locomotor activity and exploratory behavior. Compared to the Blank group, Model group rats exhibited a significant increase in total distance traveled (*p* < 0.001) and a significant decrease in immobility time (*p* < 0.01). In contrast, both the DZP (*p* < 0.01) and CC-GF (*p* < 0.05) groups showed significantly reduced total distance and significantly increased immobility time vs. the Model group (Figures 1A, B). These results indicate that DZP and CC-GF treatment suppressed abnormal excitation and alleviated anxiety in insomnia model rats, suggesting anxiolytic and sedative-hypnotic effects.

The Morris water maze test evaluated spatial exploration ability in insomnia-induced rats using escape latency as a key indicator. Compared to the control group, the model group exhibited an extremely significant increase in escape latency (*p* < 0.05). Both the DZP group and CC-GF group showed significantly reduced escape latency compared to the model group (*p* < 0.05; Figure 1C). Tracking analysis via the water maze imaging system revealed distinct behavioral patterns: control rats predominantly searched quadrant IV (original platform location) with directed trajectories, while model group rats displayed disorganized, purposeless swimming. DZP-treated rats exhibited peripheral swimming near the target quadrant with trajectories concentrated in quadrant IV, whereas CC-GF-treated rats showed similar peripheral behavior but with increased exploration between quadrants I and IV, indicating enhanced spatial exploration capacity and autonomous behavioral patterns after CC-GF intervention (Figure 1D).

Quantitative analysis of quadrant IV interactions showed that model group rats had significantly reduced entries (*p* < 0.01), average velocity (*p* < 0.05), path length, and dwell time compared to controls. All drug-treated groups demonstrated improved performance in these metrics compared to the model group (*p* < 0.05), suggesting CC-GF effectively mitigates insomnia-related anxiety/depression and exhibits anxiolytic effects through enhanced spatial memory and exploratory behavior (Figures 1E–H).

On day 3 of modeling, the model group exhibited significantly reduced serum 5-HT levels compared to the blank control group (*p* < 0.001), confirming successful establishment of the PCPA-induced insomnia model in rats. Following drug administration, the model group showed significantly lower serum levels of both 5-HT and GABA compared to the control group (*p* < 0.05). Compared to the model group, both the DZP group and CC-GF group demonstrated markedly elevated serum 5-HT levels (*p* < 0.001 and *p* < 0.001, respectively) and significantly increased GABA concentrations (*p* < 0.01), indicating effective modulation of neurotransmitter imbalances through pharmacological interventions (Figures 1I,J).

### 3.2 CC-GF effectively reverses insomnia-related gut microbiota dysbiosis as revealed by α/β diversity and species composition analysis

The community structure of gut microbiota consists of species diversity and compositional characteristics. Analyses of these structural features enable comprehensive evaluation of the biodiversity and taxonomic composition of gut microbiota under different intervention conditions. Alpha-diversity reflects within-sample microbial diversity based on species abundance and evenness, whereas beta-diversity quantifies inter-group differences in community structure and species composition via distance-based algorithms.

**Figure 2:**
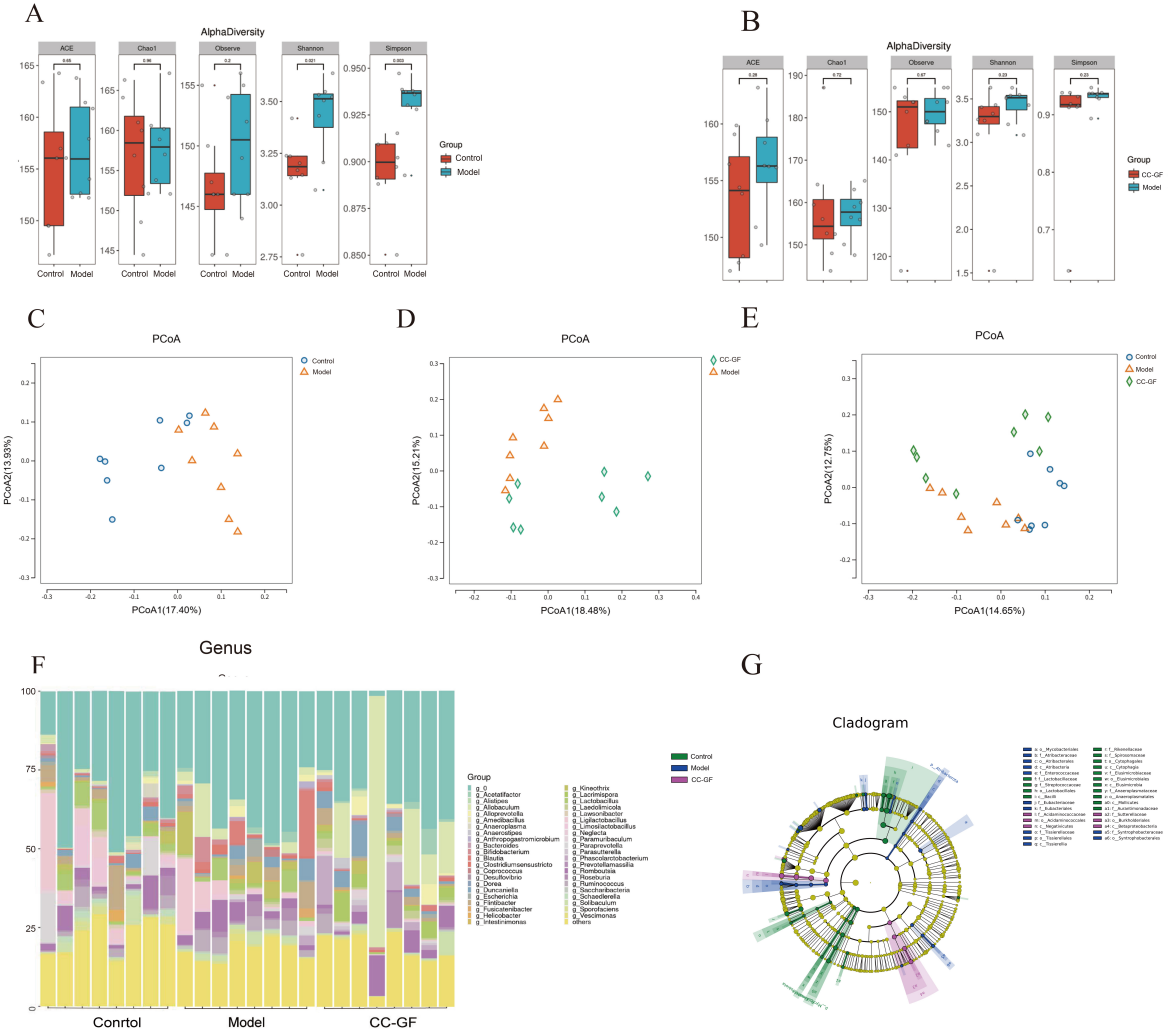
CC-GF ameliorates gut microbiota dysbiosis in PCPA-induced insomnia rats; **(A,B)** α-diversity indices (Chao1, ACE, Shannon, and Simpson) in each group; **(C–E)** β-diversity analysis based on unweighted UniFrac distance using principal coordinate analysis (PCoA); **(F)** Genus-level gut microbiota composition in each group; **(G)** Cladogram of differential intestinal microbiota among groups analyzed by LEfSe (LDA score ≥ 2). Data are presented as mean ± SEM.

To evaluate changes in gut microbial diversity and composition, we analyzed alpha-diversity, beta-diversity, and genus-level community structure. No significant differences in alpha-diversity indices including Chao1, ACE, Shannon, and Simpson were observed between the Control and Model groups, as well as between the Model and CC-GF groups (Figures 2A, B). Beta-diversity analysis using principal coordinate analysis (PCoA) based on unweighted UniFrac distances revealed obvious separation of microbial communities in the Model group from those in the Control and CC-GF groups (Figures 2C, D), while the CC-GF group mainly clustered close to the Control group (Figure 2E). These results suggest that insomnia mainly affects species richness rather than overall diversity, and CC-GF intervention facilitates the restoration of gut microbial structure to a normal physiological state, thereby effectively reversing disease-associated gut dysbiosis.

Genus-level compositional analysis demonstrated significant overall structural differences in gut microbiota among the three groups (Figure 2F). Compared with the Control group, the Model group exhibited altered microbial composition, which was partially reversed by CC-GF treatment. LEfSe analysis (LDA ≥ 2) was performed to screen differential microbial biomarkers, and the cladogram intuitively illustrated the hierarchical distribution of differential taxa from class to family level across the three groups. Taxa such as *Enterococcaceae* were enriched in the Model group; Lactobacillaceae and related taxa were dominant in the Control group; and Sutterellaceae were enriched in the CC-GF group. These findings indicate that CC-GF specifically regulates the abundance of signature microbiota at multiple taxonomic levels and re-establishes intestinal microecological homeostasis (Figure 2G).

### 3.3 Screening results of differentially expressed genes from the GEO dataset

**Figure 3:**
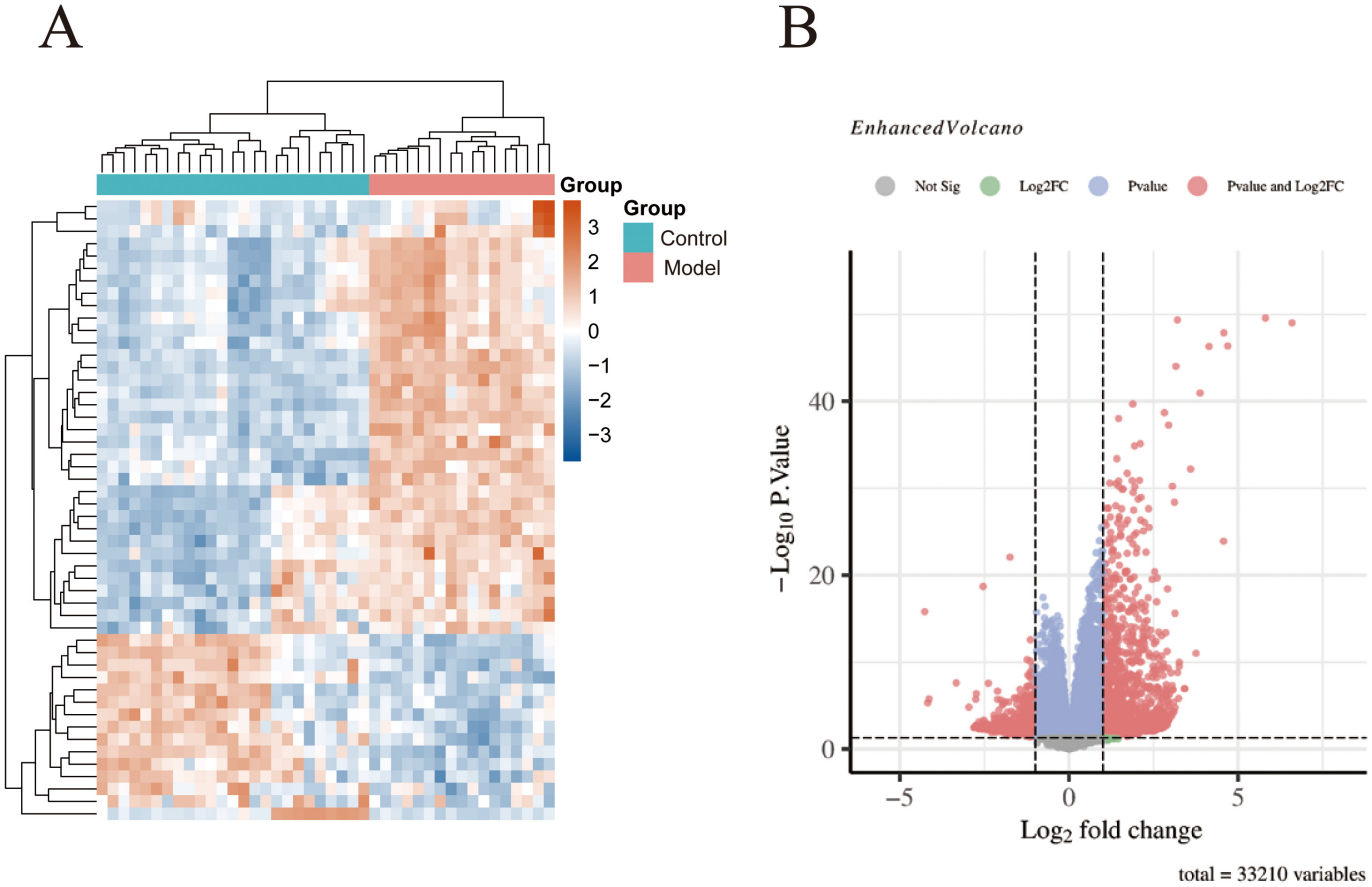
GEO differential gene screening results. **(A)** Heatmap of major DEGs, showing the differential expression patterns between samples; **(B)** Volcano plot of differentially expressed genes.

We selected the microarray dataset GSE208668 (17 insomnia cases vs. 25 healthy controls) and performed standardized data preprocessing as described in the Materials and methods Section. Differential gene expression analysis was conducted using the limma package, with the thresholds set at |log2 fold change (FC)| > 1 and *P*-value < 0.05. A total of 6,980 statistically significant differentially expressed genes (DEGs) were identified between the insomnia group and the control group. Among them, 3,316 genes were significantly upregulated, while 3,664 genes were significantly downregulated. These DEGs exhibited distinct expression patterns between the two groups and may be involved in the biological mechanisms underlying insomnia in the elderly. The overall distribution and expression profiles of the DEGs were visualized using heatmaps and volcano plots (Figures 3A, B).

### 3.4 Twenty-eight putative targets of gut microbiota metabolites for insomnia

**Figure 4:**
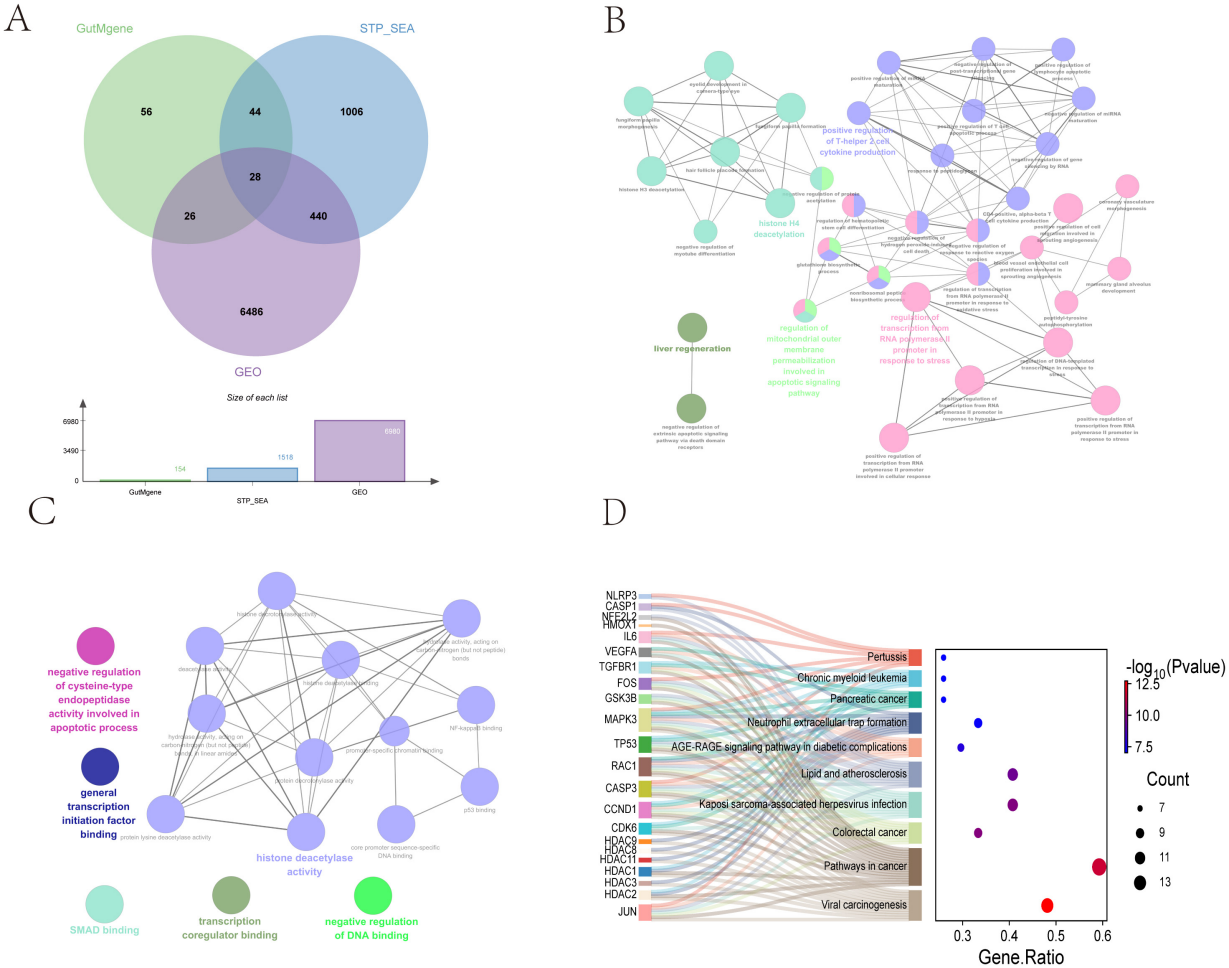
Venn diagram and GO/KEGG analysis of gut microbiota metabolites and insomnia-related targets. **(A)** Overlap of gut microbiota metabolite targets, Microbial metabolite-Host Gene, and insomnia-related targets from the GEO database; **(B)** BP enrichment analysis results for key predicted targets; **(C)** MF enrichment analysis results for key predicted targets; **(D)** KEGG pathway enrichment analysis of key predicted targets.

Based on the gut microbiota metabolites and corresponding targets identified from the Similarity Ensemble Approach and Swiss Target Prediction platforms, and by intersecting these with the microbial metabolite-host gene data, a total of 72 potential host targets regulated by gut microbiota metabolites were obtained. Simultaneously, after integration and deduplication of data from the GEO database, 6,980 insomnia-related targets were retrieved. By performing intersection analysis between these 6,980 insomnia-related targets and the 72 potential host targets regulated by gut microbiota metabolites, 28 key targets were identified, which were considered as the main predicted targets for gut microbiota metabolites regulating insomnia (Figure 4A).

### 3.5 Predicted targets are enriched in inflammation, apoptosis, and immune-related pathways

To further explore the biological functions of the key predicted genes, Gene Ontology (GO) functional enrichment analysis was performed, including two main categories: Biological Process (BP) and Molecular Function (MF). In the BP category (Figure 4B), the enriched GO terms primarily involved immune regulation, transcriptional regulation, and cell apoptosis. Representative terms included “positive regulation of T-helper 2 cell cytokine production,” “liver regeneration,” and “histone H4 deacetylation,” suggesting that key genes play an important role in immune response, cellular stress, and programmed cell death. In the MF category (Figure 4C), the enriched terms were mainly related to transcriptional regulation, histone deacetylase activity, and enzyme binding functions. Significantly enriched functions included “histone deacetylase activity,” “general transcription initiation factor binding,” and “protein lysine deacetylase activity,” indicating that key genes may participate in the disease progression through mechanisms such as transcriptional regulation and chromatin remodeling.

KEGG pathway enrichment analysis (Figure 4D) showed that the differentially expressed genes were significantly enriched in disease-related pathways, such as “Cytokine-cytokine receptor interaction,” “TNF signaling pathway,” and “NF-kappa B signaling pathway.” This suggests that the differentially expressed genes play an important role in processes such as inflammatory response, apoptosis, and immune regulation.

### 3.6 Four core therapeutic targets (CASP3, NFE2L2, HDAC1, and CCND1) identified from PPI network

**Figure 5:**
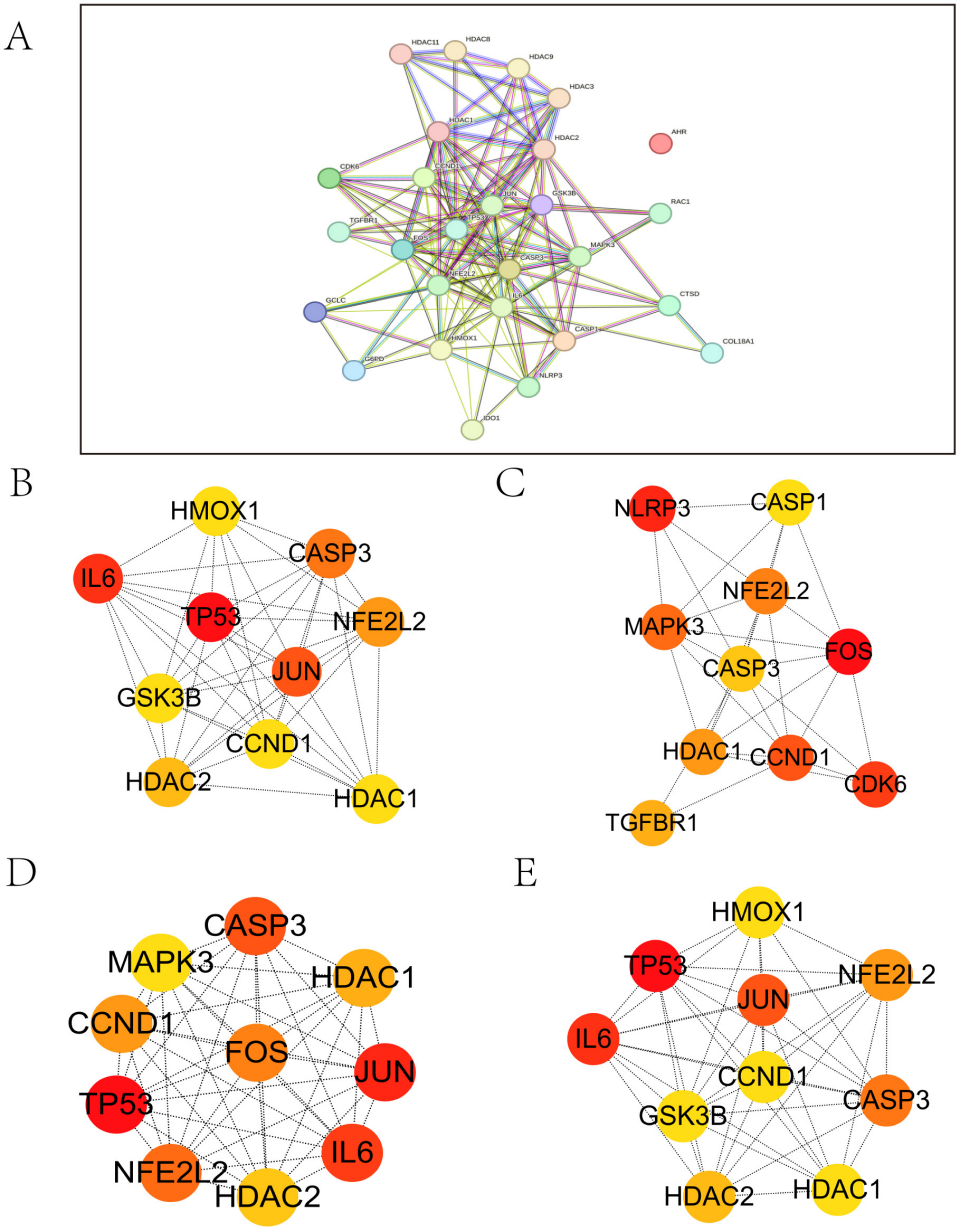
PPI network analysis and core target screening. **(A)** PPI network of key predicted targets; **(B–E)** Core targets screened based on four algorithms (Degree, DMNC, MCC, and MNC) in the CytoHubba plugin of Cytoscape, showing the top 10 targets selected by each algorithm.

**Figure 6:**
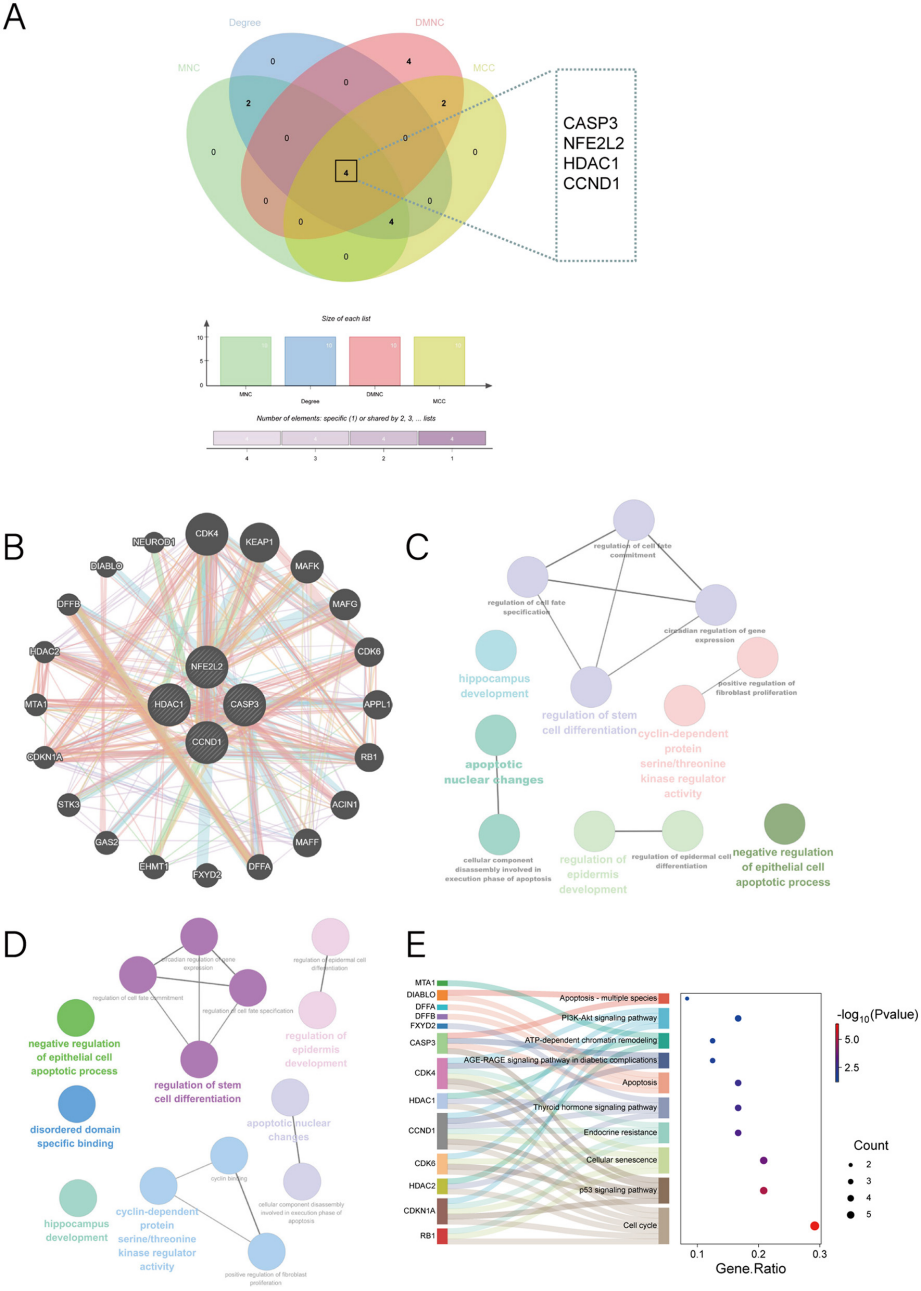
Core target screening and geneMANIA functional association of core targets. **(A)** Venn diagram showing the overlap of core targets screened by four algorithms, with the final identification of 4 core targets (CASP3, NFE2L2, HDAC1, and CCND1); **(B)** Functional association network of core targets; **(C,D)** Enrichment results of the targets in BP and MF categories; **(E)** KEGG pathway enrichment results of the targets.

To further identify the core targets playing a key role in insomnia treatment, we imported the 28 key targets into the STRING database, restricted to the species “Homo sapiens,” and set the “medium confidence > 0.4” for analysis. The results generated by STRING were then imported into Cytoscape 3.10.2 to construct the PPI network, which included 26 nodes and 140 edges (Figure 5A). Next, we used the CytoHubba plugin in Cytoscape, applying four algorithms (Degree, DMNC, MCC, and MNC) to screen for core targets (Figures 5B–E). We selected the top 10 targets from each algorithm and used a Venn diagram to identify the overlap among them. The final results revealed that four genes were identified as the core therapeutic targets for insomnia: CASP3, NFE2L2, HDAC1, and CCND1 Figure 6A.

### 3.7 GeneMANIA functional association of core targets and GO/KEGG enrichment analysis

Based on co-expression, physical interactions, and genetic associations, we added genes related to the four core targets to the gene set, resulting in a total of 24 functionally associated genes (Figure 6B). Through this process, we were able to more accurately identify potential therapeutic target genes with significant potential.

Enrichment analysis of the 24 genes was conducted for GO functional and KEGG pathway analysis. The main BP-related pathways include “regulation of stem cell differentiation” and “regulation of cell fate specification” (Figure 6C), which may be associated with neurological function and circadian rhythm disruption. Additionally, processes related to apoptosis, such as “apoptotic nuclear changes,” were also significantly enriched, suggesting that cellular stress may play a role in the development of insomnia. The MF analysis revealed significant enrichment in “cyclin binding” and “cyclin-dependent protein serine/threonine kinase regulator activity” (Figure 6D), indicating a relationship between cell proliferation and neural activity. Moreover, KEGG pathway enrichment analysis of the expanded genes corresponding to core targets demonstrated that the targets were mainly enriched in the cell cycle, p53 signaling pathway, cellular senescence, and other related pathways. These pathways are involved in key pathological processes of insomnia including neuronal apoptosis, cell cycle regulation, neuroendocrine regulation, oxidative stress, and chromatin remodeling, suggesting that CC-GF may improve insomnia by regulating the above pathways (Figure 6E).

### 3.8 Network and intersection analysis reveals four core gut microbiota for CC-GF against insomnia

**Figure 7:**
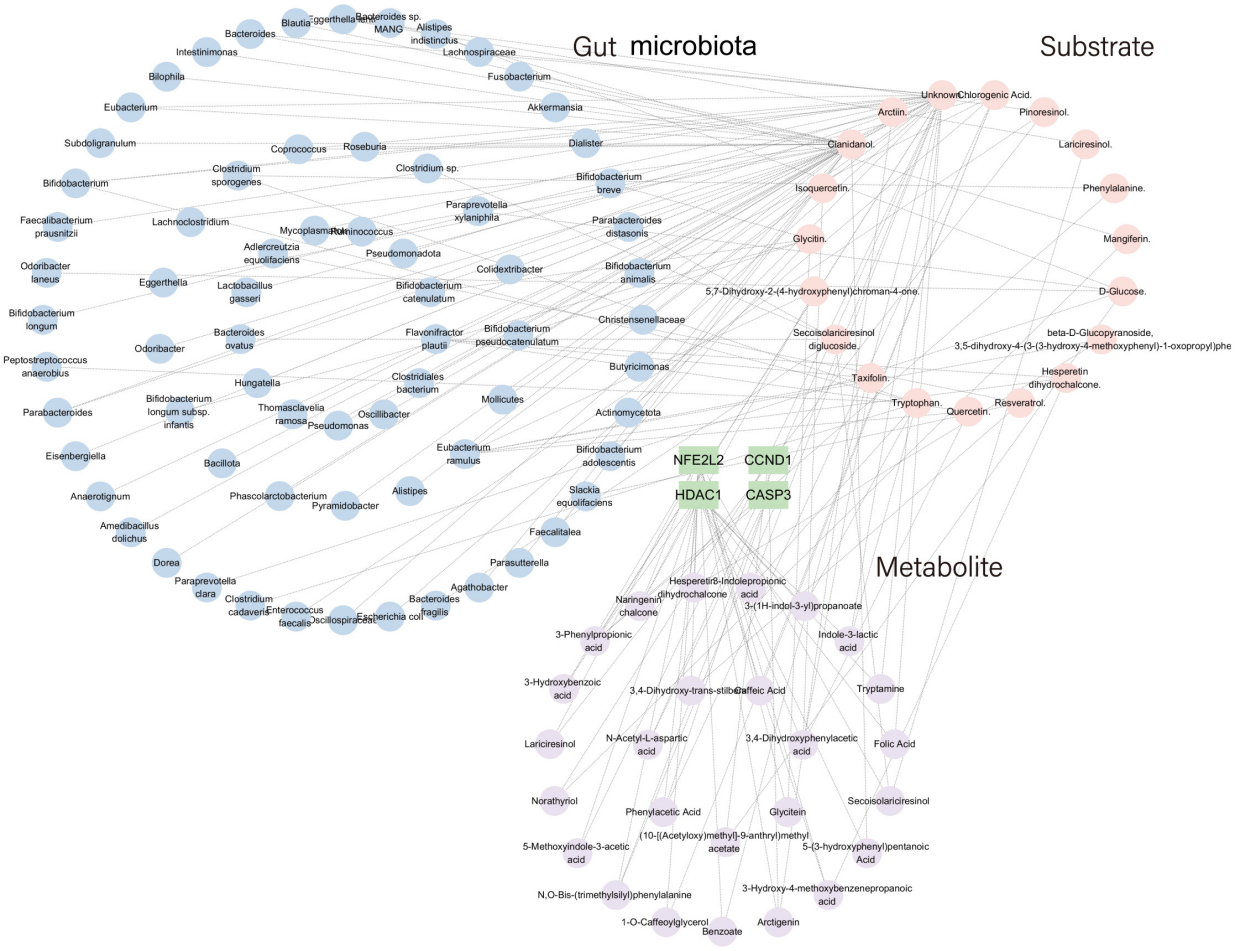
“Gut microbiota-metabolites-substrate-core targets” network diagram.

To clarify the relationship between the predicted core targets and the gut microbiota, we constructed a “Gut Microbiota-Metabolites-Substrate-Core Targets” network (Figure 7), which illustrates the complex interactions among these components. The network includes 119 nodes (comprising 70 gut microbiota species, 26 gut microbiota substrates, 4 targets, and 19 metabolites) and 149 edges. We identified the gut microbiota that directly regulate the key predicted targets.

**Figure 8:**
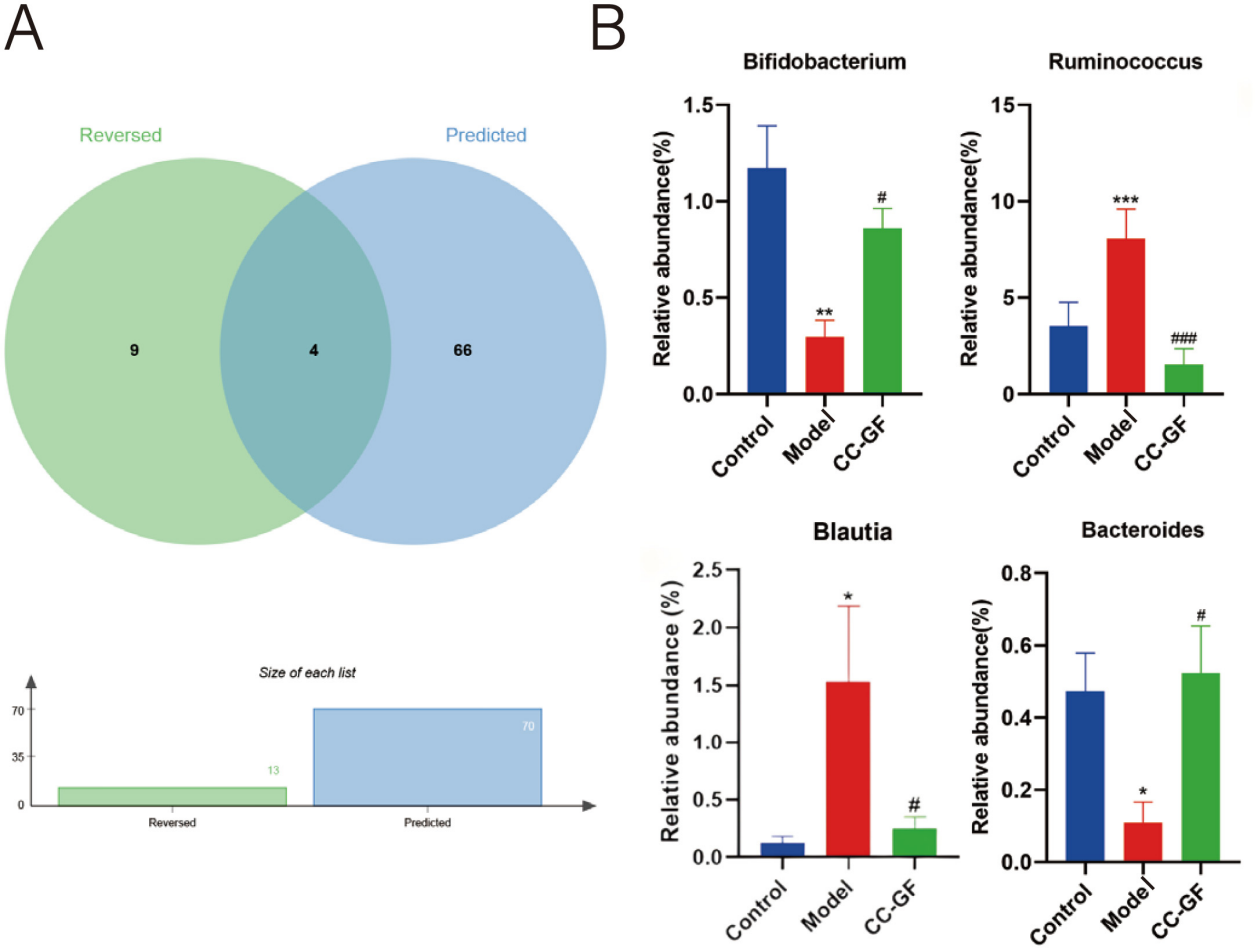
Core gut microbiota screening. **(A)** Venn diagram showing the intersection of predicted disease-related gut microbiota and differential gut microbiota in the insomnia model, identifying four key gut microbiota species; **(B)** Changes in the relative abundance of the four key gut microbiota species after CC-GF intervention. Data are mean ± SD.*p < 0.05, **p < 0.01, and ***p < 0.001 (Model vs. Control); ^#^*p* < 0.05, ^###^*p* < 0.001 (CC-GF vs. Model).

The predicted disease-associated gut microbiota was intersected with the differential microbiota observed in the insomnia animal model (Supplementary Table 1; Figure 8A), resulting in key gut microbiota species: *Bifidobacterium, Bacteroides, Ruminococcus*, and *Blautia*. After CC-GF treatment, significant changes in the abundance of these four key microbiota species were observed, and they were considered the core gut microbiota through which CC-GF exerts its anti-insomnia effect.

### 3.9 *In vivo* validation of core gut microbiota and analysis of genus-level changes

Genus-level differential analysis revealed significant dysregulation of several gut microbiota species in the insomnia model, which showed a clear reversal trend following CC-GF intervention. Among them, *Bifidobacterium, Bacteroides, Ruminococcus*, and *Blautia* exhibited consistent and biologically meaningful changes during both disease progression and drug intervention.

Compared to the control group, the relative abundance of *Bifidobacterium* and *Bacteroides* was significantly reduced in the insomnia model group, while *Ruminococcus* and *Blautia* showed significant increases, suggesting that the gut microbiota homeostasis is disrupted in the insomnia state. After CC-GF intervention, the abundance of *Bifidobacterium* and *Bacteroides* significantly increased, while the abnormal elevation of *Ruminococcus* and *Blautia* was notably suppressed, indicating that CC-GF has a role in reshaping the gut microbiota imbalance associated with insomnia (Figure 8B). Comprehensive analysis revealed that the aforementioned genera all exhibited a consistent trend of “disease-related changes—reversal by drug intervention,” and thus were further selected as the core gut microbiota through which CC-GF exerts its anti-insomnia effect.

### 3.10 Core microbiota-metabolite-target and gene regulatory networks

**Figure 9:**
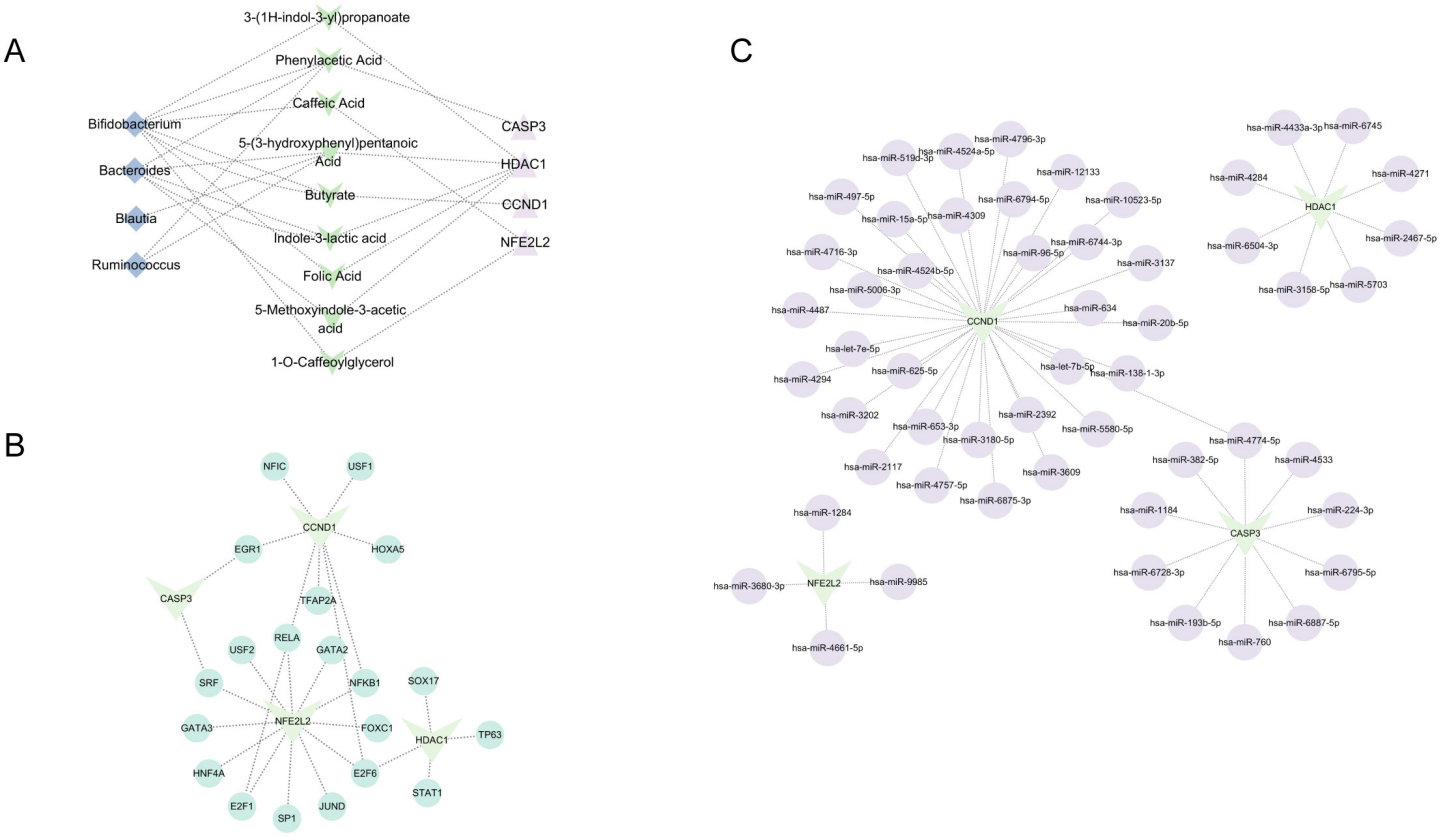
Network diagram construction. **(A)** “Core gut microbiota–metabolites–core targets” diagram; **(B)** Gene-transcription factor network diagram; **(C)** Gene-miRNA network diagram.

To explore the potential mechanism by which CC-GF regulates insomnia through gut microbiota, we further constructed a “core gut microbiota-metabolite-core target” regulatory network (Figure 9A). The network contained 19 nodes, including 4 gut microbiota, 9 metabolites and 4 core targets, as well as 25 edges. A total of nine metabolites that mediate the regulatory effect of core gut microbiota on core targets were screened out and identified as key metabolites, namely Phenylacetic Acid, 5-Methoxyindole-3-acetic acid, Indole-3-lactic acid, 3-(1H-indol-3-yl)propanoate, 5-(3-hydroxyphenyl)pentanoic Acid, Folic Acid, Butyrate, Caffeic Acid and 1-O-Caffeoylglycerol. Regulatory network analysis generated interaction networks between genes and transcription factors (Figure 9B) and between genes and miRNAs (Figure 9C). CASP3 was predicted to interact with 20 miRNAs and 2 transcription factors, while CCND1 was predicted to interact with 33 miRNAs and 5 transcription factors. NFE2L2 interacted with 7 miRNAs and 12 transcription factors, and HDAC1 interacted with 8 miRNAs and 4 transcription factors. The gene-transcription factor network analysis indicated that the transcription factors E2F1 and NFKB1 could simultaneously regulate the expression of CCND1 and NFE2L2, while E2F6 could simultaneously regulate the expression of HDAC1 and NFE2L2. The gene-miRNA network analysis revealed that hsa-miR-4774-5p could simultaneously regulate the expression of both CASP3 and CCND1.

### 3.11 Molecular docking

**Figure 10:**
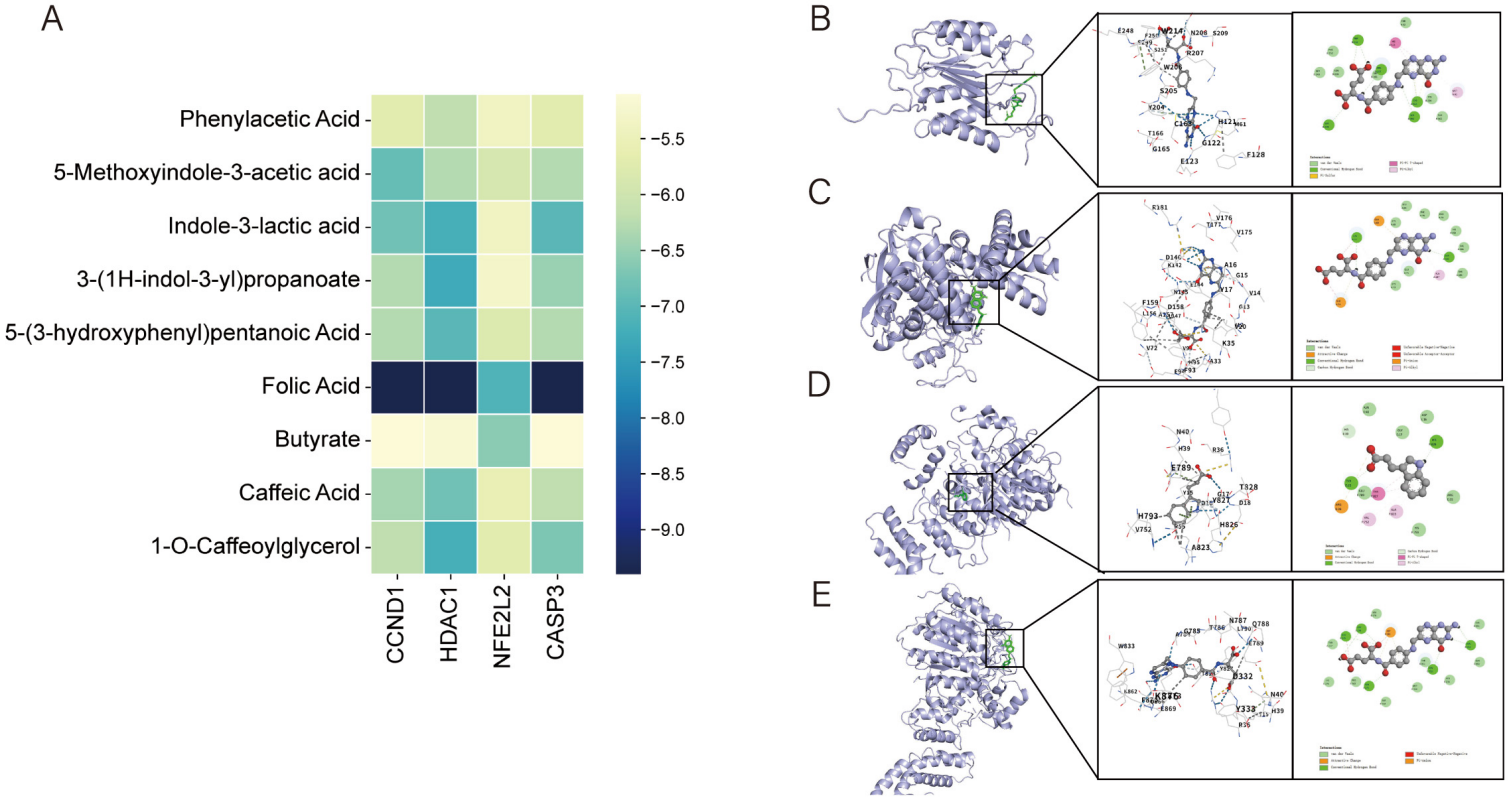
Molecular docking results. **(A)** Molecular docking binding energy diagram; **(B)** CASP3-Folic Acid; **(C)** CCND1-Folic Acid; **(D)** HDAC1-3-(1H-indol-3-yl)propanoate; **(E)** HDAC1-Folic Acid.

Molecular docking results showed that the nine compounds selected in this study exhibited varying degrees of binding affinity with the four core targets (CCND1, HDAC1, NFE2L2, and CASP3; Figure 10A), with average binding energies ranging from −5.13 to −8.83 kcal/mol. This suggests that these compounds may participate in the improvement of insomnia mediated by the gut microbiota by targeting the regulation of these proteins. Among them, Folic Acid exhibited the lowest average binding energy with the four core targets (−8.83 kcal/mol), indicating a broad-spectrum and strong binding advantage. The top four lowest binding energies in the molecular docking were CASP3-Folic Acid, CCND1-Folic Acid, HDAC1-3-(1H-indol-3-yl) propanoate, and HDAC1-Folic Acid (Figure 10B–E), which were further visualized for analysis.

### 3.12 Molecular dynamics simulation

**Figure 11:**
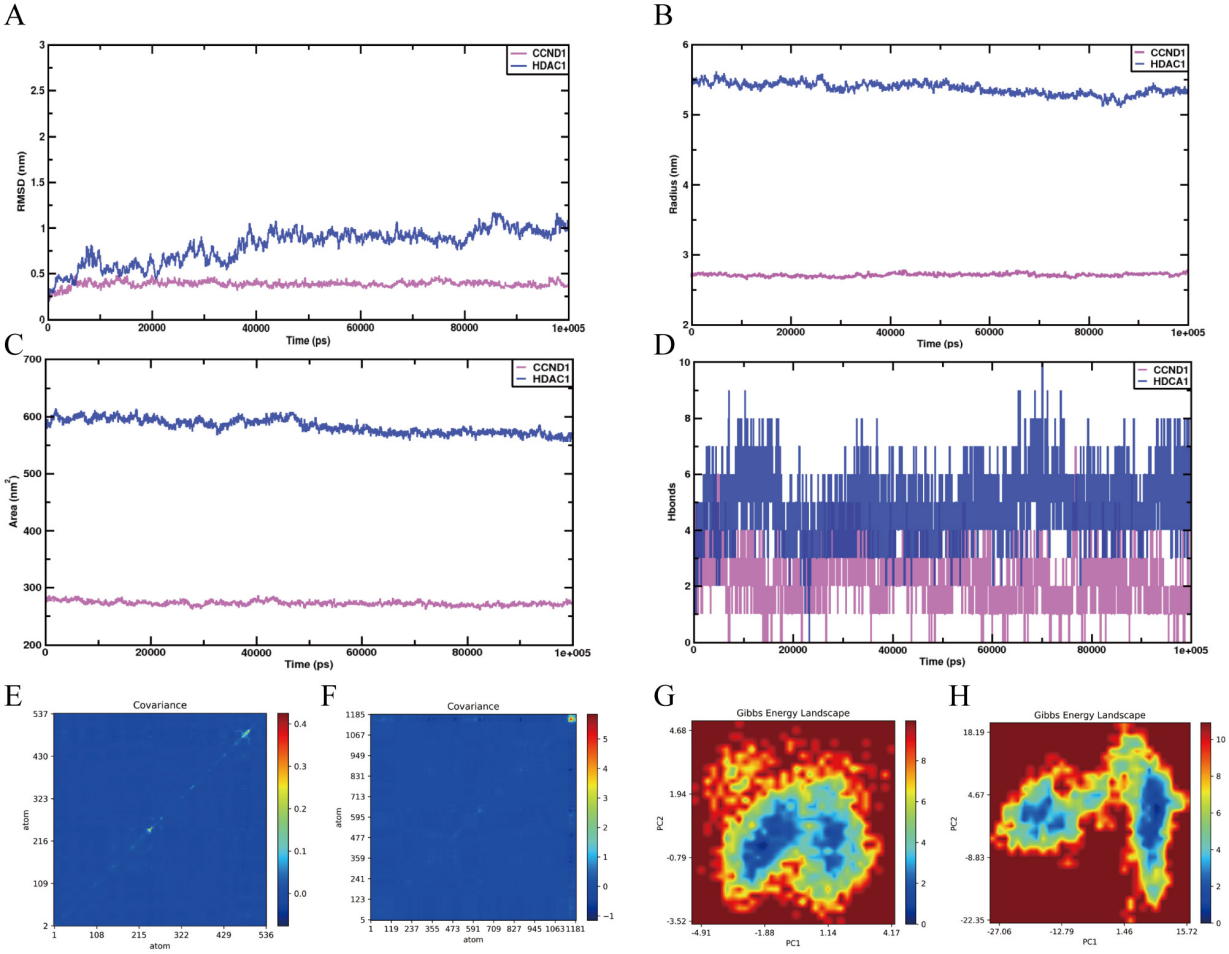
Molecular dynamics simulation results and structural analysis. **(A)** Changes in RMSD values reveal differences in structural stability between the CCND1 and HDAC1 complexes during simulation; **(B)** Changes in Rg show the variations in the molecular radius of the two complexes; **(C)** Changes in SASA demonstrate the fluctuations in the solvent-accessible surface area of the two complexes; **(D)** Changes in the number of hydrogen bonds illustrate the fluctuations in hydrogen bond counts for the two complexes throughout the simulation; **(E)** Covariance map of CCND1, showing the interactions between atoms in the CCND1 molecular system; **(F)** Covariance map of HDAC1, displaying the interatomic relationships within the HDAC1 molecular system; **(G)** Free energy landscape of CCND1, depicting the dynamic energy states of CCND1 during simulation; **(H)** Free energy landscape of HDAC1, illustrating the changes in different energy states of HDAC1 throughout the simulation.

For the most stable binding complexes, CASP3-Folic Acid and CCND1-Folic Acid were further subjected to molecular dynamics simulations. The results showed that both CCND1 and HDAC1 formed stable complexes with their ligands. RMSD analysis (Figure 11A) showed that the structure of the CCND1 complex fluctuated minimally throughout the simulation, maintaining a low level, indicating its high stability. The HDAC1 complex, after initial minor fluctuations, stabilized, suggesting that it maintained good conformational consistency after adaptation. Rg analysis (Figure 11B) showed that both complexes had minimal fluctuations in compactness during the simulation, with no significant expansion, maintaining high compactness. SASA results (Figure 11C) indicated that the surface exposure area of both proteins remained steady, with no significant conformational changes observed. Hydrogen bond analysis (Figure 11D) revealed that the number of hydrogen bonds in the CCND1 complex fluctuated slightly (ranging from 0 to 7), while the hydrogen bond count in the HDAC1-ligand complex was significantly higher. Although some fluctuations were observed, the number of hydrogen bonds remained at a higher level, reflecting the formation of a more abundant and stable hydrogen bond network between the ligand and HDAC1, which also corresponded to the rapid stabilization of the HDAC1 complex after initial fluctuations. Further principal component analysis (PCA) results (Figures 11E–H) provided deeper insights into the conformational dynamics of the two complexes.

For the CCND1 complex, the atomic motion covariance matrix (Figure 11E) is predominantly dark blue, with only weak bright signals observed locally, indicating very low correlation between atomic motions and highly restricted conformational fluctuations. The corresponding Gibbs free energy landscape (Figure 11G) shows a concentrated and continuous low-energy basin, containing only one major dominant conformational cluster, with a narrow energy core region. This reflects that the conformational changes of the CCND1 complex during the simulation are confined to a very small space, remaining consistently stable in a single low-energy conformation. For the HDAC1 complex, the covariance matrix (Figure 11F) is mainly dark blue, indicating low covariance, but significant high-covariance signals are observed in certain regions, suggesting the existence of cooperative movement among local atomic groups (such as in the ligand-binding region or flexible loops). The Gibbs free energy landscape (Figure 11H) shows two relatively independent low-energy conformational clusters, with a broader conformational space distribution, but both clusters remain in a low-energy state. This suggests that after initial fluctuations, the HDAC1 complex formed two stable conformational subgroups, reflecting the adaptability of the structure while maintaining good conformational consistency. In summary, both CCND1 and HDAC1 complexes exhibited good structural stability and binding reliability during the simulation.

## 4 Discussion

Despite advances in insomnia neurobiology and pharmacological therapy, current treatments are limited by insufficient efficacy and adverse-effect risks, highlighting the need for new insights into its pathogenesis and management strategies. Emerging studies demonstrate that gut microbiota and their metabolites modulate central nervous system function through the gut-brain axis, providing a novel perspective for insomnia research (Doenyas et al., 2025; Shi et al., 2025; Liu et al., 2024). Microbiota-derived metabolites (such as short-chain fatty acids and tryptophan derivatives) serve as signaling molecules that regulate neurotransmitter synthesis, oxidative stress and neuroinflammation to mediate sleep homeostasis (Dos Santos and Galiè, 2024; Gao et al., 2020). Nevertheless, mechanistic studies focusing on gut microbial metabolites in insomnia are still scarce, and how specific metabolites modulate host targets during insomnia pathogenesis remains poorly defined.

This study first validated the therapeutic efficacy of CC-GF using a PCPA-induced acute insomnia rat model. As a classic insomnia-inducing agent, PCPA irreversibly inhibits tryptophan hydroxylase (TPH), the rate-limiting enzyme for 5-HT synthesis, to reduce central 5-HT levels and trigger insomnia-like phenotypes (Koe and Weissman, 1966; Jouvet, 1999; Ye et al., 2025). Intraperitoneal PCPA (300 mg/kg for 2 consecutive days) stably induces insomnia characterized by prolonged sleep latency, shortened total sleep time, and decreased hypothalamic 5-HT and GABA levels, which has been widely validated in previous studies (Ye et al., 2025; Ren et al., 2020; Suzuki et al., 1981; Si et al., 2022). Consistent with existing reports, our model rats exhibited typical insomnia-related manifestations including irritability, social withdrawal, weight loss and abnormal fecal properties, confirming successful model construction.

Open-field test (OFT) results indicated insomnia-induced hyperactivity and anxiety-like behaviors in model rats, consistent with prior PCPA-related studies (Kraeuter et al., 2019; Ren et al., 2020; Wang et al., 2024). CC-GF intervention significantly alleviated such abnormal behaviors, as reflected by reduced total travel distance and recovered immobility time, suggesting its sedative, anxiolytic and hypnotic effects. Sleep disturbance commonly impairs spatial learning and memory, as verified by Morris Water Maze (MWM) assays (Zielinski et al., 2013; McKenna et al., 2019). Here, CC-GF effectively reversed insomnia-induced spatial cognitive dysfunction by shortening escape latency and improving target-quadrant performance, supporting its protective role against sleep-related memory deficits, which is consistent with the effects of herbal-derived compounds reported previously (Shen et al., 2020).

Neurotransmitters 5-HT and GABA are key regulators of the sleep-wake cycle, and their deficiency is closely associated with insomnia pathogenesis (Li et al., 2025; Zhu et al., 2024). We found that CC-GF markedly increased serum 5-HT and GABA levels with efficacy comparable to diazepam, in accordance with previous herbal medicine studies (Bian et al., 2021). Taken together, CC-GF ameliorates insomnia-induced physical, behavioral, cognitive and neurotransmitter disorders, which provides a solid pharmacodynamic basis for further exploring its anti-insomnia mechanism via the gut-brain axis.

This study systematically explored the mechanism linking gut microbiota metabolites to insomnia, aiming to clarify the role of the “gut microbiota-metabolite-target” axis in insomnia pathogenesis. Via integrated network pharmacology and 16S rRNA sequencing, we identified four core targets (CASP3, NFE2L2, HDAC1, and CCND1), characterized insomniac gut microbiota dysbiosis, and constructed a “core gut microbiota-metabolite-core target” regulatory network, with folic acid as the key metabolite. Using the food-medicine homologous combination CC-GF as an intervention, we found it reversed insomnia-related gut microbiota and metabolite abnormalities, providing experimental evidence for the “gut microbiota-metabolite” axis in insomnia regulation. Subsequent sections discuss these core targets, gut microbiota alterations, key metabolites, and their interconnections.

Through network pharmacology, 28 potential targets related to insomnia were predicted, which are involved in gut microbiota and its metabolites. GO enrichment analysis indicated that these genes might regulate disease progression through immune signaling, epigenetic regulation, and apoptosis pathways. KEGG pathway analysis suggested that these genes are primarily enriched in inflammation and immune regulation-related pathways. Through PPI network analysis and multi-algorithm screening, CASP3, NFE2L2, HDAC1, and CCND1 were ultimately identified as the core targets for the intervention of insomnia by gut microbiota and its associated metabolites. These four core targets represent four interconnected key dimensions in the pathogenesis of insomnia: oxidative stress (NFE2L2), apoptosis (CASP3), epigenetic regulation (HDAC1), and circadian rhythm disruption (CCND1).

NFE2L2 (Nrf2), a key intracellular redox-sensing transcription factor, regulates multiple antioxidant genes to maintain cellular oxidative homeostasis (Ngo and Duennwald, 2022). Sleep and oxidative stress exhibit a bidirectional relationship: sleep deprivation elevates systemic oxidative stress, and Nrf2, as a redox sensor and regulator, is critical for sleep homeostasis, implying NFE2L2 modulates sleep quality via oxidative stress regulation (Davinelli et al., 2024). Consistently, acute sleep deprivation upregulates Nrf2-related antioxidant genes in mammals, further validating Nrf2 as a core mediator of sleep deprivation responses (Wang et al., 2023). Notably, our experimental results showed that CC-GF treatment restored serum 5-HT and GABA levels and alleviated anxiety-like behaviors in model rats, which may be partially attributed to NFE2L2-mediated enhancement of antioxidant capacity and reduction of central oxidative stress. Caspase-3 (CASP3), the central executioner of neuronal apoptosis (D'Amelio et al., 2012), is activated in rat brains under acute REM sleep deprivation, accompanied by increased caspase-3 protein levels. Sleep deprivation may trigger neuronal apoptosis through caspase-3-dependent pathways and impair sleep regulation (Somarajan et al., 2016), thus CASP3 activation contributes to insomnia pathogenesis (Milazzo et al., 2020). In our study, CC-GF-induced recovery of 5-HT and GABA may further protect sleep-related neurons by inhibiting CASP3-mediated apoptotic cascades, thereby relieving insomnia-related behavioral and cognitive disorders. HDAC1, a histone deacetylase family member (English et al., 2024), participates in epigenetic regulation of sleep. Sleep deprivation disturbs histone acetylation-deacetylation balance via HDACs (HDAC1/2), indicating HDAC1 mediates sleep-related gene expression alterations (Gaine et al., 2018). CC-GF may modulate HDAC1-driven epigenetic modifications to promote the expression of 5-HT- and GABA-synthetic genes, further improving sleep homeostasis. As a cell-cycle protein regulated by circadian CLOCK/BMAL1, CCND1 expression changes under circadian disruption, altering cell-cycle progression and RB phosphorylation, which links CCND1 to insomnia-associated neurobiological events (Lee et al., 2019). Further gene-transcription factor and gene-miRNA network analyses identified key regulatory factors controlling these core targets, providing potential molecular pathways for CC-GF-mediated anti-insomnia effects. Moreover, CC-GF-restored neurotransmitter balance may stabilize circadian rhythm via regulating CCND1-related signaling pathways.

Network pharmacology constructed an integrated insomnia associated network containing 70 gut microbiota species, 26 substrates and 4 core targets. By matching differential microbiota from 16S rRNA sequencing between blank control and insomnia model groups with the 70 predicted taxa, four core gut microbiota were screened. In model rats, *Bifidobacterium* and *Bacteroides* were markedly depleted, whereas *Ruminococcus* and *Blautia* were enriched. CC-GF intervention restored the abundance of *Bifidobacterium* and *Bacteroides* and suppressed the overgrowth of *Ruminococcus* and *Blautia*. Pathologically, *Bifidobacterium* and *Bacteroides* are regarded as beneficial taxa. *Bifidobacterium longum* improves sleep quality by modulating 5 HT and GABA levels (Gavzy et al., 2023; Patterson et al., 2024). As commensal bacteria, *Bacteroides* maintain gut homeostasis and indirectly regulate sleep (Zafar and Saier, 2021). By contrast, *Blautia* and *Ruminococcus* are potential harmful bacteria. *Blautia* is significantly increased in insomnia populations, contributing to gut dysbiosis (Wang et al., 2025). Altered abundance of *Ruminococcus* and Ruminococcaceae in sleep disorder models is linked to short chain fatty acid metabolism and gut brain axis signaling (Li et al., 2021).

Using the “core gut microbiota-metabolite-core target” regulatory network, nine key metabolites were screened: phenylacetic acid, 5-methoxyindole-3-acetic acid, indole-3-lactic acid, 3-(1H-indol-3-yl)propanoate, 5-(3-hydroxyphenyl)pentanoic acid, folic acid, butyrate, caffeic acid, and 1-O-caffeoylglycerol. Molecular docking showed folic acid exhibited the optimal broad-spectrum targeting ability, with an average binding energy of −8.83 kcal/mol for four core targets. As a central mediator connecting gut microbiota to host insomnia-related pathways under CC-GF intervention, folic acid was identified as the core metabolite.

As a major intestinal folate producing genus, *Bifidobacterium* differs from other gut microbes dependent on exogenous folate; specific strains synthesize folate *de novo* via the shikimate pathway (generating p aminobenzoic acid) and folate biosynthetic cascade. Previous studies have verified that *Bifidobacterium adolescentis* DSM 18352 and MB 239 significantly increase fecal folate concentrations in healthy adults, and folate producing bifidobacteria enable colonic endogenous folate synthesis (Pompei et al., 2007a,b; Strozzi and Mogna, 2008).

Folic acid status strongly correlates with sleep quality. By participating in one carbon metabolism, it modulates the synthesis of key sleep regulating neurotransmitters including serotonin and melatonin, thus affecting the sleep wake cycle. Moderate serum folate levels reduce sleep disturbance risk via regulating neurotransmitter production and circadian rhythms. Additionally, folate exhibits a nonlinear U shaped association with sleep, as both deficiency and excess impair sleep health (An et al., 2023; Tu et al., 2025). Consistently, the 8 year longitudinal EPISONO study confirmed that sub threshold folate levels correlate with worsened insomnia (Cavalcante-Silva et al., 2025), indicating folic acid exerts therapeutic effects mainly in folate deficiency related insomnia.

Folic acid interacts closely with the four core targets. It exerts epigenetic regulation by modulating HDAC1 expression and activity: folate intervention inhibits HDAC1 expression and activity while upregulating histone acetylation, and high dose folate further suppresses HDAC1 transcription and total HDAC activity to regulate neuronal gene expression via enhanced histone acetylation (Gao et al., 2025; Clark et al., 2021). Together with vitamin B12, folic acid synergistically modulates PPARγ and the apoptosis related gene CASP3. Folate deficiency elevates homocysteine (Hcy) to induce apoptosis, whereas folate intervention upregulates PPARγ mRNA and downregulates CASP3 transcription—effects strengthened by vitamin B12 co supplementation. In neural stem cells, folate deficiency triggers Hcy accumulation to upregulate CASP3 expression (Zhang et al., 2009; Lv et al., 2013). Folic acid also regulates NFE2L2. It activates the Nrf2 antioxidant pathway by upregulating NFE2L2 and suppresses HMGB1 mediated inflammation, alleviating central nervous oxidative stress and inflammatory injury, which underlies its insomnia regulating function (Du et al., 2021). Moreover, folate modulates CCND1 expression via miRNA to mediate neural development, markedly downregulating gga mir let 7k 5p in the cerebral cortex to increase CCND1 levels (Khoshkenar et al., 2025).

In summary, intestinal *Bifidobacterium* modulates systemic one carbon metabolism via folate synthesis to regulate neurotransmitter production. Meanwhile, folate targets four core genes (NFE2L2, CASP3, HDAC1, and CCND1) involved in oxidative stress, apoptosis, epigenetic modification and circadian rhythm regulation, respectively.

Indole 3 lactic acid, 3 (1H-indol-3-yl)propanoate, and 5 methoxyindole 3 acetic acid show average binding energies of −6.35 to −6.60 kcal/mol against the four core targets, defined as secondary active compounds. These typical indole derivatives are generated by gut microbiota via tryptophan metabolism (Miao et al., 2025).

Multiple tryptophan derived gut microbial metabolites participate in gut brain axis signaling. Acting as chemical messengers alongside classic neurotransmitters including serotonin and GABA, they cross the blood brain barrier to modulate central nervous system function, or activate enteroendocrine cells and the enteric nervous system to stimulate vagal transmission to the brainstem, thus regulating sleep related physiological processes (Cheng et al., 2025). Notably, *Bifidobacterium* produces indole 3 lactic acid from tryptophan (Yong et al., 2024); indole 3 lactic acid further modulates downstream antioxidant responses via the aryl hydrocarbon receptor (AhR) NRF2 axis, indicating its potential regulation of NFE2L2 related pathways (Jia et al., 2025).

1-O-caffeoylglycerol, a glycerol ester derivative of caffeic acid, are both phenolic compounds (Kang et al., 2009). Dietary polyphenols such as caffeic acid regulate neurotransmitter systems (e.g., GABA, serotonin) and modulate gut-microbiota-central nervous system crosstalk to affect sleep (Pérez-Jiménez et al., 2023). Butyrate, a short-chain fatty acid, acts on hypothalamic sleep-regulating centers via the gut-brain axis (Szentirmai et al., 2019). Phenylacetic acid, a gut-microbiota-derived metabolite from phenylalanine metabolism, regulates inflammation and neural function through phenylacetylglutamine (PAGln) production, suggesting its indirect sleep-modulating role via the gut-brain axis (Krishnamoorthy et al., 2024).

Although this study preliminarily elucidates the core pathway by which CC-GF regulates insomnia via the gut-brain axis, several limitations exist. The GSE208668 dataset used for target prediction was mainly derived from elderly individuals, and inherent differences in metabolism, physiological status and gut microecology between elderly and young/middle-aged populations may somewhat affect the universality of the identified targets. We only used a PCPA-induced acute insomnia rat model, which cannot fully mimic the complex etiology of clinical chronic insomnia. Meanwhile, mechanistic exploration was merely based on network pharmacology and molecular docking predictions, lacking *in vitro* cellular and *in vivo* functional validation to confirm the causal relationship of the gut microbiota-metabolite-target axis. In addition, strain-level taxonomic analysis and dynamic monitoring of post-intervention microbiota were not performed, limiting in-depth exploration of how gut microbiota modulate insomnia. Notably, we did not directly detect folic acid levels or their correlation with folate-producing gut microbiota. Nevertheless, changes in *Bifidobacterium* abundance and strong binding affinity from molecular docking indirectly support this regulatory axis, which requires further validation via metabolomics studies. Future research will employ strain-level metagenomic sequencing to screen functional strains associated with insomnia and folate metabolism, and will also recruit age-matched clinical insomnia cohorts to verify the stability and applicability of the “gut microbiota-metabolite-target” axis across different age groups. Meanwhile, clinical observational and small-sample intervention studies will be conducted to clarify the correlations among gut microbiota composition, key metabolites and sleep quality, providing evidence for the clinical application of CC-GF.

## 5 Conclusion

In this study, an integrated strategy combining network pharmacology and 16S rRNA sequencing was employed to elucidate the role of the “gut microbiota-metabolite-target” axis in insomnia pathogenesis. Four core targets—CASP3, NFE2L2, HDAC1, and CCND1—were identified, corresponding to four pathological dimensions: oxidative stress, apoptosis, epigenetic regulation, and circadian rhythm disruption. 16S rRNA sequencing revealed significant gut microbiota dysbiosis under insomniac conditions, characterized by decreased abundance of beneficial bacteria (*Bifidobacterium* and *Bacteroides*) and increased abundance of potentially harmful bacteria (*Ruminococcus* and *Blautia*). A “core gut microbiota-metabolite-core target” regulatory network was constructed, identifying nine key metabolites. Among these, folic acid exhibited the strongest binding affinity to the four core targets, suggesting that a “*Bifidobacterium*-folic acid-target” axis may represent a potential pathway in insomnia regulation. Using the food-medicine homologous herbal formula CC-GF as an intervention example, we demonstrated its ability to reverse insomnia-related gut microbiota dysbiosis and metabolite abnormalities, providing experimental evidence for the involvement of the gut microbiota-metabolite axis in insomnia regulation. Collectively, this study offers a new theoretical basis for understanding insomnia from a gut microecological perspective and lays an experimental foundation for future intervention strategies targeting gut microbiota metabolites.

## Data Availability

The data presented in the study are deposited in the China National GeneBank Sequence Archive (CNSA, CNGB), accession number CNP0009492.
