# Supplementary material for: The gut microbiota-metabolite-target axis in insomnia: network pharmacology and gut microbiota profiling insights into pathogenesis and intervention with Cinnamomi Cortex-Gardeniae Fructus
Source: Front Microbiol. 2026 Jun 29;17:1830736. doi: 10.3389/fmicb.2026.1830736 (PMC13357922; doi:10.3389/fmicb.2026.1830736)
Supplement: Supplementary file 1 [file Table_1.DOCX]

**Supplementary Table S1: Gut microbiota genera altered by CC-GF treatment**

| Rank | Genus | Model vs. Control | CC-GF vs. Model |
| --- | --- | --- | --- |
| 1 | *Escherichia* | ↑ *** | ↓ ** |
| 2 | *Solibaculum* | ↑ * | ↓ * |
| 3 | *Ruminococcus* | ↑ * | ↓ *** |
| 4 | *Saccharibacteria* | ↑ *** | ↓ ** |
| 5 | *Kineothrix* | ↑ ** | ↓ ** |
| 6 | *Blautia* | ↑ * | ↓ * |
| 7 | *Acetatifactor* | ↑ *** | ↓ * |
| 8 | *Clostridiumsensu stricto* | ↑ *** | ↓ ** |
| 9 | *Lacrimispora* | ↓ * | ↑ *** |
| 10 | *Lawsonibacter* | ↓ * | ↑ *** |
| 11 | *Bifidobacterium* | ↓ * | ↑ * |
| 12 | *Paramuribaculum* | ↓ * | ↑ *** |
| 13 | *Ligilactobacillus* | ↓ *** | ↑ * |
| 14 | *Flintibacter* | ↑ | ↓ * |
| 15 | *Limosilactobacillus* | ↑ | ↓ *** |
| 16 | *Alloprevotella* | ↓ | ↑ ** |
| 17 | *Laedolimicola* | ↓ | ↑ * |
| 18 | *Bacteroides* | ↓ | ↑ ** |
| 19 | *Romboutsia* | ↓ | ↑ * |
| 20 | *Phascolarctobacterium* | ↑ | ↓ ** |

Statistical significance: * *p* < 0.05, ** *p* < 0.01, *** *p* < 0.001; ↑, indicates significantly increased relative abundance; ↓, indicates significantly decreased relative abundance.
